# The complete mitochondrial genome of freshwater mussel *Pronodularia japanensis* (Gonideinae, Unionidae, Unionida) from Tochigi Prefecture, Japan, and its phylogenetic analysis

**DOI:** 10.1080/23802359.2020.1730726

**Published:** 2020-02-27

**Authors:** Yohei Fukata, Masayuki Iigo

**Affiliations:** a Department of Applied Biological Chemistry, School of Agriculture, Utsunomiya University, Tochigi, Japan;; b Department of Applied Life Science, United Graduate School of Agricultural Science, Tokyo University of Agriculture and Technology, Tokyo, Japan;; c Center for Bioscience Research and Education, Utsunomiya University, Tochigi, Japan;; d Center for Optical Research and Education, Utsunomiya University, Tochigi, Japan;; e Center for Weed and Wildlife Management, Utsunomiya University, Tochigi, Japan

**Keywords:** *Pronodularia japanensis*, Unioninae, freshwater mussel, Illumina sequence, molecular phylogenetic analysis

## Abstract

We have sequenced the female-type (F-type) complete mitochondrial genome of *Pronodularia japanensis* (Gonideinae, Unionidae, Unionida, Bivalvia) from Tochigi Prefecture, Japan. The complete F-type mitochondrial genome (16,803 bp; LC505454) contains 13 protein-coding genes, 2 rRNA genes, and 22 tRNA genes. Molecular phylogenetic analyses using complete F-type mitochondrial genomes of 56 Unionida species revealed the phylogenetic position of *P. japanensis* in Unionidae. This study should be basic data to investigate the genetic diversity in this species.

The freshwater mussels of the family Unionidae are benthic bivalves that inhabit rivers and lakes widely. Unionidae consists of four subfamilies: Gonideinae, Ambleminae, Anodontinae, and Unioninae (Huang et al. 2013). Unionidae are endangered species around the world. *Pronodularia japanensis* (Gonideinae, Unionidae, Unionida, Bivalvia) inhabits in Japan, but the population of this species has been decreasing. Therefore, it is necessary to conserve this species.

Mitochondrial genome sequences are valuable resources for systematics and conservation biological studies. However, the complete mitochondrial genome of *P. japanensis* has not been reported. Therefore, in this study, we have sequenced the F-type complete mitochondrial genome of *P. japanensis* and molecular phylogenetic analyses were performed using complete mitochondrial genomes of Unionidae.

*Pronodularia japanensis* (#UU-SBD-Unio-01) was collected in Tochigi Prefecture, Japan (N36.8, E140.1). Genomic DNA Extraction from the mantle, library preparation, next-generation sequencing, trimming of nucleotide sequences, *de novo* sequence assembly and annotation of mitochondrial genome were performed as previously described (Fukata and Iigo 2019). Local BLAST search using the mitochondrial genome of *Unio japanensis* (AB055625) identified the complete mitochondrial genome of *P. japanensis* from Tochigi, Japan (circular, 16803 bp). The results of BLAST search at NCBI (https://blast.ncbi.nlm.nih.gov/Blast.cgi) showed that the mitochondrial genome is the F-type. The complete mitochondrial genome contains 13 protein-coding genes, 2 rRNA genes, and 22 tRNAs. The sequence has been submitted to DDBJ/EMBL/Genbank with an accession number of LC505454.

Molecular phylogenetic analysis was performed using the complete mitochondrial genomes of Unionoida. We included complete mitochondrial genomes of Margaritiferidae (*Gibbosula crassa*, NC_037942; *Margaritifera marocana*, NC_034911; *Cumberlandia monodonta,* NC_034846) as an outgroup. The sequences were aligned by ClustalW and molecular phylogenetic tree was constructed by the maximum likelihood method using MEGA X (Kumar et al. 2018). Bootstrap analysis was performed with 1000 replications.

The molecular phylogenetic tree (Figure 1) revealed the following relationships: Anodontinae/Unioninae and Ambleminae/Gonideinae are sister subfamilies. *Pronodularia japanensis* is the closest to *Potomida littoralis* (NC_030073).

**Figure 1.**
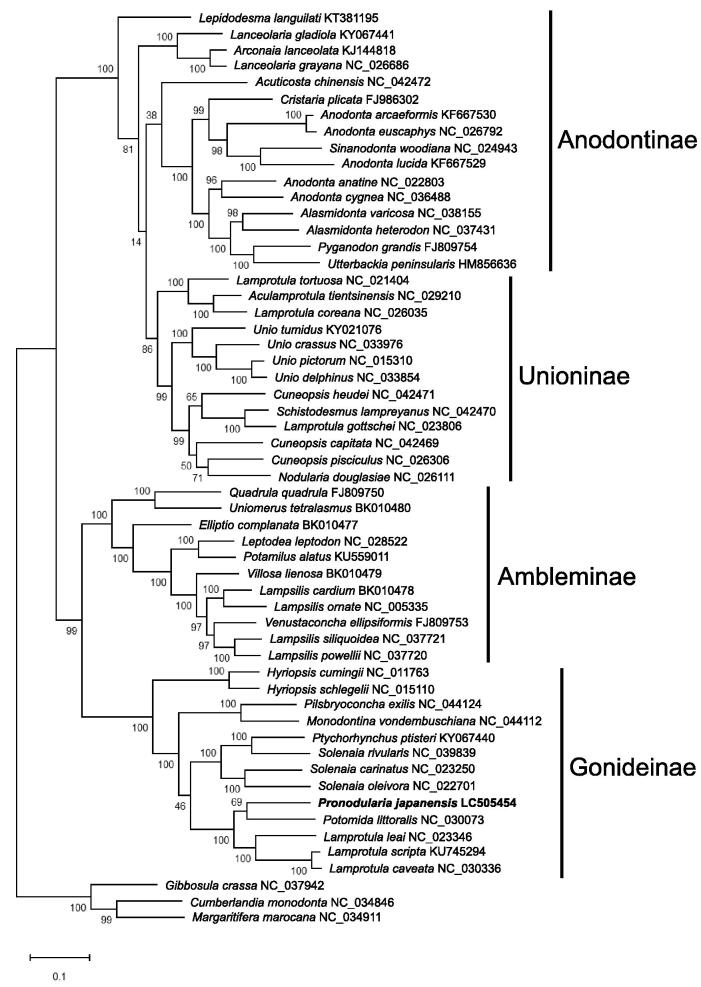
Molecular phylogenetic tree (Maximum likelihood method) using F-type complete mitochondrial genomes of 56 Unionoida species including *P. japanensis* from Tochigi, Japan. The numbers above the branch meant bootstrap value (1000 replicates). Leaf names were presented as species names and accession number. The sequence determined in this study (LC505454) is in bold.

Former molecular phylogenetic analyses of the Unionidae based on partial mitochondrial genomes showed inconsistent relationships among studies (Bolotov et al. 2017; Lopes-Lima et al. 2017; Huang et al. 2018; Wu et al. 2019). However, the results of our study are consistent with the previous result based on complete mitochondrial genomes of Unionidae (Wang et al. 2018).

In conclusion, our study described the complete mitogenome of *P. japanensis*, and defined its phylogenetic position among Unionidae. This research would facilitate further investigations of the evolution of Unionidae. This research would also provide valuable information for investigating the genetic diversity of local populations of *P. japanensis*.
